# Automated real-time surveillance of *Bithynia* snails using a comparative YOLO based approach for liver fluke host detection

**DOI:** 10.1038/s41598-026-43387-x

**Published:** 2026-03-24

**Authors:** Thannaree Jenwithee, Thirayu Meererksom, Yanin Limpanont, Banchob Sripa, Thewarach Laha, Apiporn T. Suwannatrai

**Affiliations:** 1 Department of Parasitology, Faculty of Medicine, Khon Kaen University, Khon Kaen, Thailand; 2 Translational Medicine Program, Institute of Medicine, Suranaree University of Technology, Nakhon Ratchasima, Thailand; 3 Department of Social and Environmental Medicine, Faculty of Tropical Medicine, Mahidol University, Bangkok, Thailand; 4 Department of Tropical Medicine, Faculty of Medicine, Khon Kaen University, Khon Kaen, Thailand

**Keywords:** *Bithynia* species, *Opisthorchis viverrini*, YOLO, Deep Learning, Automated classification, Disease surveillance, Human-AI Collaboration, Object detection metrics, Biological techniques, Computational biology and bioinformatics, Ecology, Ecology

## Abstract

*Bithynia* species serve as obligate intermediate hosts for *Opisthorchis viverrini*, a Group 1 carcinogen endemic to Southeast Asia and the primary etiological agent of cholangiocarcinoma. Accurate identification of morphologically similar *Bithynia* species is critical for effective disease surveillance. This study systematically evaluates four You Only Look Once (YOLO) models—YOLOv5, YOLOv8, YOLOv10, and YOLOv11—for automated detection and classification of *Bithynia* species. Models were trained on 4,204 images containing 8,559 annotated specimens of *Bithynia funiculata*, *B. siamensis goniomphalos*, and *B. s. siamensis*. Model performance was assessed through comparative analyses of YOLO architectures and direct benchmarking against five human experts. YOLOv10 demonstrated the highest classification accuracy (98.7%), robust performance across diverse environmental conditions, and computational efficiency (model size: 31.9 MB; processing speed: 4.54 FPS). Our analysis reveals a fundamental complementarity between AI and human expertise: humans achieved perfect detection accuracy, while the artificial intelligence model provided superior classification of detected specimens (74.5% for YOLOv10 and 48.3% for human experts, respectively). These findings indicate that integrating human detection with automated classification in hybrid human–AI workflows could substantially improve diagnostic accuracy and throughput. This study establishes a technological foundation for scalable surveillance systems to support control of *O. viverrini* transmission in resource-limited endemic regions.

**Supplementary Information:**

The online version contains supplementary material available at 10.1038/s41598-026-43387-x.

## Introduction

The genus *Bithynia* (Family: Bithyniidae) represents one of the most medically important groups of freshwater gastropods worldwide. While these snails are integral components of aquatic ecosystems^1^, their primary significance lies in serving as obligate intermediate hosts for food-borne trematodes responsible for substantial global disease burden^2,3^. Infections caused by food-borne trematodes affect over 40 million people globally, with around 750 million at risk^4,5^. The genus exhibits wide distribution across Europe, Asia, and Africa, comprising approximately 130 recognized species^6^. Of particular medical significance are the 25 Oriental species that serve as the exclusive intermediate hosts for regionally endemic and highly pathogenic trematodes^6,7^.

Medically significant liver flukes exhibit distinct regional associations with specific intermediate snail hosts. In East Asia, particularly China, Korea, and northern Vietnam, *Bithynia fuchsiana* functions as the primary intermediate host for *Clonorchis sinensis*, a liver fluke affecting more than 15 million people^8–11^. Across Northern Asia, Eastern Europe, and Western Siberia, *Opisthorchis felineus* transmission is mainly associated with *Bithynia tentaculata*, *B. troscheli*, and *B. leachii*^12,13^. In Southeast Asia, *Opisthorchis viverrini*, a Group 1 carcinogen and the leading cause of cholangiocarcinoma in the region is primarily transmitted by *Bithynia siamensis* (including subspecies *B. s. siamensis* and *B. s. goniomphalos*) and *B. funiculata*^14,15^. These host-parasite associations are well established across Thailand, Laos, Cambodia, Myanmar, and central Vietnam^16–19^.

The taxonomy of *Bithynia* snails in Thailand was initially described by Brandt^20^, recognizing two subgenera, *Gabbia* and *Digoniostoma*, each comprising three species. Within *Bithynia* (*Digoniostoma*) *siamensis*, two subspecies were identified: *B. s. siamensis* and *B. s. goniomphalos*. *Gabbia* was later elevated to genus status^21,22^. Chitramvong^21,22^ subsequently expanded the classification of Thai Bithyniidae, identifying ten species distributed among several genera: *Bithynia funiculata*, *B. siamensis goniomphalos*, *B. s. siamensis*, *Gabbia wykoffi*, *G. pygmaea*, *G. erawanensis*, *Wattebledia crosseana*, *W. siamensis*, *W. baschi*, and *Hydrobioides nassa*. Species differentiation is based on morphological features including shell structure, internal organ anatomy, and radular cusp patterns^23,24^. However, taxonomic uncertainty persists, as several species within Bithyniidae remain incompletely characterized morphologically^23,24^.

Recent ecological investigations have revealed crucial range expansions that surpass traditional geographic distributions, with *B. s. goniomphalos* and *B. s. siamensis* establishing new populations in northern and southern regions^32–34^. This observed expansion can be attributed to several factors, including enhanced hydrological connectivity^35,36^, climate-driven habitat alterations^37^, and anthropogenic disturbances^38^. These distributional shifts, coupled with the morphological complexity of *Bithynia* species, particularly their similarity to sympatric confamilial species such as *H. nassa*^39^, and *W. crosseana*^40^, pose significant challenges for accurate field identification and effective disease surveillance. Although conventional molecular techniques are reliable, both morphological and molecular approaches^41,42^ are too time-consuming and impractical for rapid, large-scale, or on-site detection. Consequently, there is an urgent need for automated, real-time identification technologies to support effective surveillance across these expanding and overlapping ranges.

Recent advances in computer vision driven by deep learning^43^ have enhanced automated detection capabilities^44^. The You Only Look Once (YOLO) algorithm represents a paradigm shift in object detection, employing end-to-end learning through a single convolutional neural network that simultaneously localizes objects and classifies them within images^45^. This unified approach eliminates the computational overhead of traditional multi-stage detection pipelines, enabling real-time processing capabilities essential for field applications^46^.

The YOLO framework has evolved rapidly, with successive generations demonstrating increased accuracy and computational efficiency. Its application in ecological surveillance began with baseline models such as YOLOv4 for gastropod recognition^47^. YOLOv5 has been successfully applied to detect medically significant species^48^ and has shown effectiveness in complex medical diagnostics such as stroke lesion detection^49^, while YOLOv8 has demonstrated improved performance in challenging scenarios involving small, occluded objects in complex environments^50^. Current state-of-the-art versions, YOLOv10 and YOLOv11, have achieved enhanced performance in critical medical applications including bone fracture detection^51^, real-time tumor analysis^52^, and Alzheimer’s classification^53^.

Despite significant advances in deep learning for object detection and the demonstrated potential of YOLO architectures for gastropod identification, comprehensive performance assessments of state-of-the-art YOLO models for detecting *Bithynia* species remain limited. To address this gap, the present study systematically evaluates and compares the performance of four key YOLO model versions (YOLOv5, YOLOv8, YOLOv10, and YOLOv11)^54–57^ in detecting and classifying *Bithynia* species, a critical step toward understanding and mitigating the transmission dynamics of *O. viverrini*. By identifying the most suitable YOLO model for* Bithynia* classification in field settings, this research establishes a foundation for automated surveillance technologies aimed at strengthening public health efforts in *O. viverrini*-endemic regions.

## Materials and methods

### Study area and sample collection

**Fig. 1:**
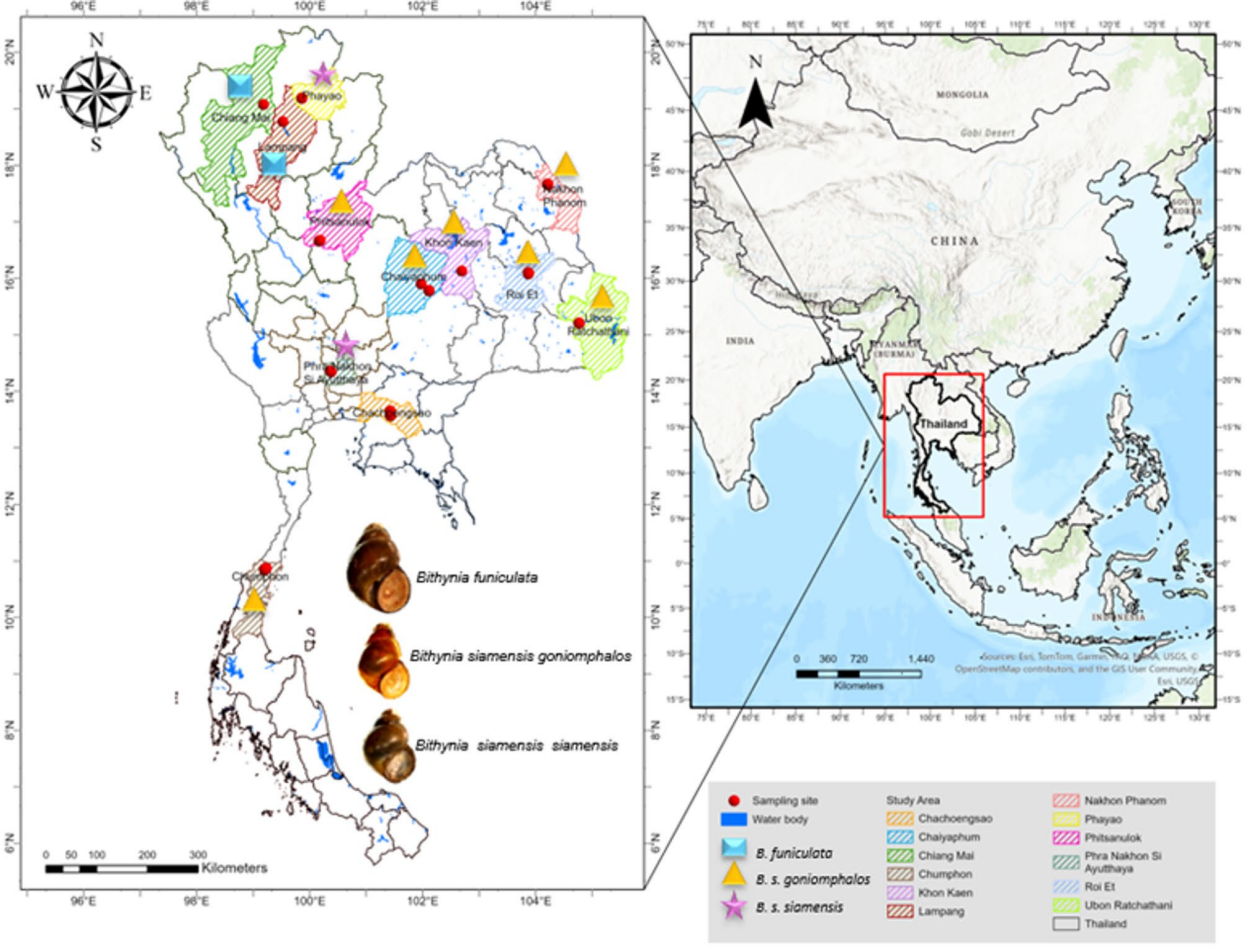
Geographical distribution of snail sampling sites in Thailand. Red circles denote sampling sites, with species-specific symbols defined in the legend. Map created via ArcGIS (ESRI Inc., Redlands, CA, USA).

Freshwater snails, with emphasis on medically significant *Bithynia* species and other co-occurring freshwater taxa, were collected between 1 September 2023 and 22 October 2024 from 47 sites across four major regions of Thailand (Fig. 1). Samples were obtained from natural habitats, including paddy fields, canals, ponds, and irrigation systems. At each site, snails were sampled from 5 to 10 stations using a standardized protocol. Manual collection was performed by two individuals at each station, consisting of a 10-minute search supplemented by hand scooping in areas with dense aquatic vegetation^58^. Collected specimens were placed in labeled plastic bags and transported to the Department of Parasitology, Faculty of Medicine, Khon Kaen University. Species identification was conducted based on morphological characteristics using Brandt’s taxonomic keys (1974)^20^, with all identifications verified by expert malacologists.

### Dataset for model development and evaluation

#### Image acquisition

The total study collection comprised 4,601 high-resolution (3024 × 4032 pixels) color images captured with an Apple iPhone 12 Pro Max mounted on a tripod. The acquisition protocol included capturing specimens against a non-reflective, white matte background as well as within their natural habitat to ensure data diversity representing field conditions.

#### Dataset preparation and partitioning

**Fig. 2:**
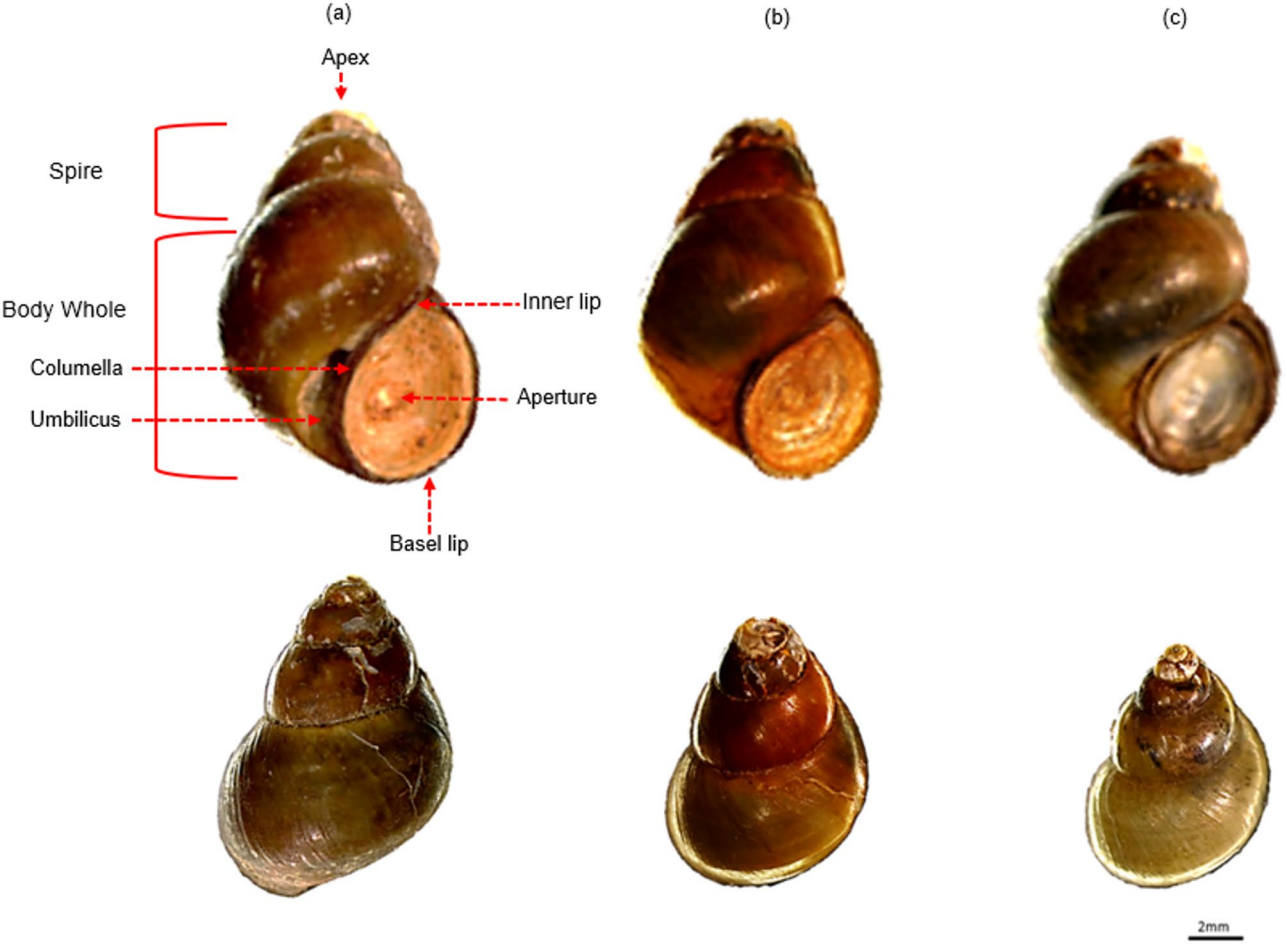
The three target *Bithynia* species. Specimens include *Bithynia funiculata* (**a**), *B. siamensis goniomphalos* (**b**), and *B. s. siamensis* (**c**) comparing distinguishing characteristics. *B. funiculata* (**a**) features a funnel-shaped umbilicus, while *B. s. goniomphalos* (**b**) possesses a long conic spire. *B. s. siamensis* (**c**) is characterized by a sharp apex and very narrow umbilicus. Scale bar = 2 mm.

The primary dataset used for model development consisted of 4,204 images with a total of 8,559 ground-truth bounding box annotations across four classes: *Bithynia funiculata* (Bf; *n* = 1,867), *Bithynia siamensis goniomphalos* (Bsg; *n* = 3,016), *Bithynia siamensis siamensis* (Bss; *n* = 3,084), as illustrated in Fig. 2, and an additional “Unknown” class (*n* = 592). All annotations were created using the Roboflow platform^59^ and independently verified by expert malacologists to ensure annotation accuracy.

#### Handling of the unknown class

The “Unknown” class included specimens that could not be reliably assigned to any of the three target species due to morphological ambiguity or incomplete visual information. This class was incorporated during training to reduce overfitting to known classes and to improve model robustness under real-world conditions. All models were trained using balanced sampling across all four classes.

During evaluation, predictions assigned to the “Unknown” class were recorded but excluded from quantitative performance metrics, which were calculated only for the three medically relevant species, following established open-set object detection practices^60,61^. Low confidence “Unknown” detections (confidence < 0.25) were filtered out during qualitative visualization.

#### Human–AI benchmark set

A separate set of 180 images was strictly reserved as the Human–AI Benchmark Set. These images were held out for final performance evaluation and remained completely unseen throughout all model development and training stages to prevent any potential bias in model selection.

### YOLO architectures

This study benchmarks the performance of four widely used YOLO architectures—YOLOv5, YOLOv8, YOLOv10, and YOLOv11 ^54,55,57,62^ —for the automated detection and classification of *Bithynia* spp. snails. To ensure that the comparison emphasizes architectural differences rather than model scale, the medium (‘m’) variant of each architecture was selected, thereby controlling for scale-dependent performance variations and enabling a fair comparative assessment.

All evaluated models share a common architectural paradigm comprising three primary components: (i) a backbone for hierarchical multi-scale feature extraction from input images, (ii) a neck for feature pyramid construction and cross-scale feature fusion, and (iii) a head for final bounding box regression and class probability prediction. Despite this shared foundation, each model incorporates distinct architectural enhancements, which are described in detail below.

**Fig. 3:**
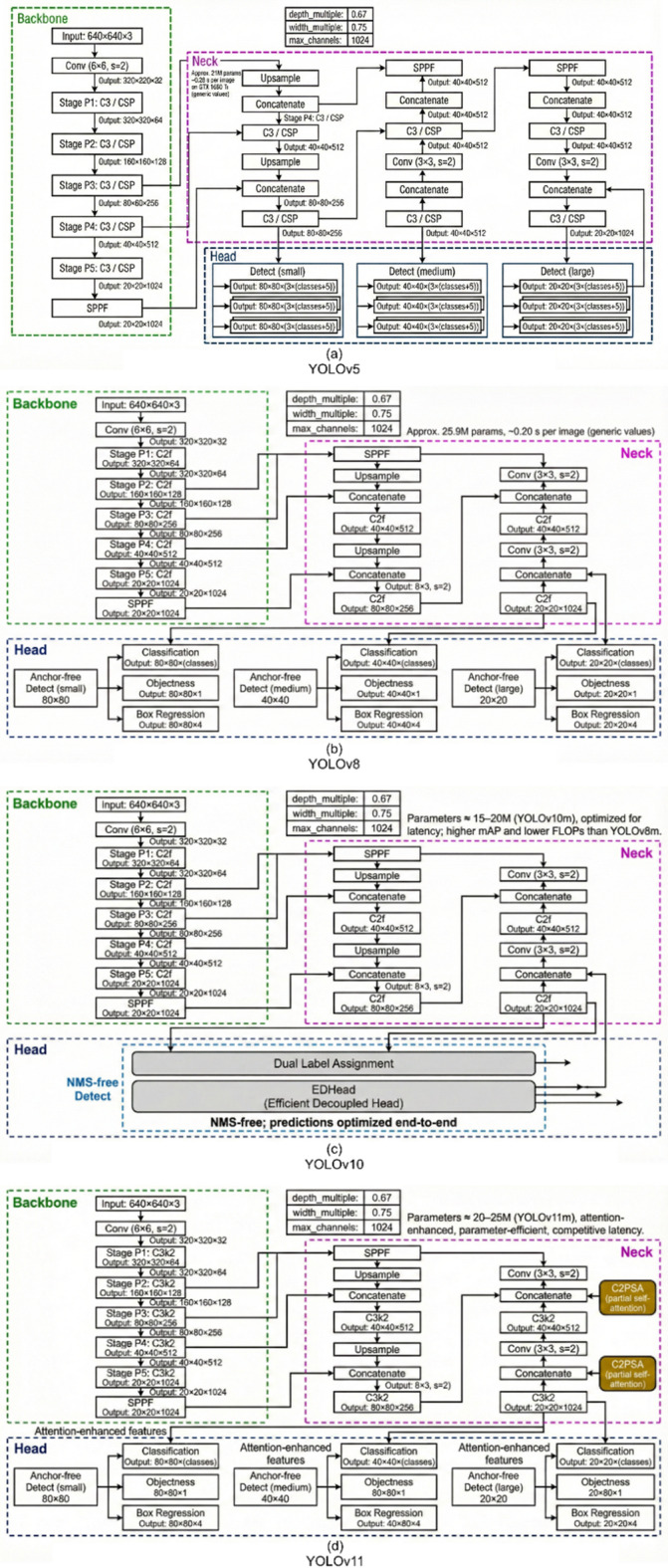
The neural architecture diagrams of (**a**) YOLOv5, (**b**) YOLOv8, (**c**) YOLOv10, and (**d**) YOLOv11.

*YOLOv5m* (Baseline Model, Fig. 3a) serves as the performance baseline for this study. Its architecture features a CSP (Cross Stage Partial) backbone and a PA-net neck, representing a foundational balance between accuracy and computational efficiency. With approximately 21.2 million parameters, the YOLOv5m variant serve as the benchmark for both predictive performance and inference, which we seek^54^.

*YOLOv8* (Contemporary Reference, Fig. 3b) enhances this baseline by introducing architectural modifications to improve efficiency. By introducing an anchor-free head and a more performant C2f module (replacing the YOLOv5 SPPF (Spatial Pyramid Pooling Fast) module), the YOLOv8m variant (approx. 25.9 million parameters) offers improved accuracy. It serves as the primary contemporary model against which the newest speed-focused optimizations are compared^55^.

*YOLOv10* (End-to-End Latency Optimization, Fig. 3c) marks a fundamental shift toward optimizing end-to-end latency. It achieves this by eliminating the Non-Maximum Suppression (NMS) post-processing step through a novel dual label assignment strategy, substantially reducing computational bottlenecks in real-time applications. This NMS-free approach, combined with a lightweight classification head and Partial Self-Attention (PSA), allows the YOLOv10-M variant to reduce inference time drastically. Its authors report that it surpasses YOLOv8m in accuracy while simultaneously reducing parameters and latency, directly addressing key deployment bottlenecks in portable surveillance systems^56^.

*YOLOv11* (Attention-Based Efficiency, Fig. 3d) pursues efficiency through advanced architectural components rather than NMS elimination. The introduction of novel C3k2 and C2PSA (Convolutional 2-layer Partial Self-Attention) attention blocks allows the YOLOv11m to achieve greater predictive power from a more compact model. It is designed to deliver superior accuracy to YOLOv8m with approximately 22% fewer parameters, offering an alternative strategy for achieving high-speed, low-resource detection^57^.

### Model training and implementation

#### Computational environment

All model training and evaluation were performed on a workstation equipped with an AMD Ryzen 5 4600 H processor, 8 GB of DDR4 RAM, and an NVIDIA GeForce GTX 1650 Ti GPU (4 GB VRAM). To ensure a consistent and reproducible computational environment, all experiments were conducted using the specific software and library versions detailed in Table 1.

**Table 1:** Software environment and computational specifications.

Component	Version	Purpose
Python	3.11.9	Core programming language
PyTorch	1.10.0	Deep learning framework
CUDA	11.3	GPU acceleration
OpenCV	4.5.4	Image processing and augmentation

#### Training protocol

This study systematically evaluated four medium-sized object detection architectures: YOLOv5m, YOLOv8m, YOLOv10m, and YOLOv11m. To ensure a direct and fair comparison, all models were fine-tuned on a custom *Bithynia* sp. snail dataset using a standardized training protocol. Each architecture was initialized with official weights pre-trained on the COCO dataset to leverage transfer learning^63^.

Training was conducted for 300 epochs with a batch size of 32, and all input images were uniformly resized to 640 × 640 pixels. To establish a rigorously controlled performance baseline derived solely from the curated dataset, no data augmentation techniques were applied. A consistent set of core hyperparameters, including the AdamW optimizer and a cosine learning rate scheduler, was used across all experiments to ensure stable convergence; detailed settings are provided in Table 2. All YOLO models were trained using the Ultralytics native Python API with identical optimizer configurations, learning rate schedules, and hyperparameter settings.

**Table 2:** Standardized training hyperparameters.

Parameter	Value	Rationale
Epochs	300	Sufficient for convergence across all architectures
Batch Size	32	Optimal for 4GB VRAM without gradient accumulation
Input Resolution	640 × 640 pixels	Standard for YOLO models; balance speed/accuracy
Optimizer	AdamW	Recommended for vision transformers and modern networks
Learning Rate	0.001	Initial rate with cosine annealing schedule
Weight Decay	0.0001	L2 regularization to prevent overfitting
Momentum	0.937	Default for YOLO framework stability
Random Seed	42	Ensures reproducible results across runs
Mixed Precision	Enabled (AMP)	Accelerates training; reduces memory overhead
Data Augmentation	None (baseline)	To establish performance based purely on curated dataset

Training Framework: Ultralytics-based models (YOLOv8, v10, v11) were implemented via their native Python API, YOLOv5 was trained using its traditional command-line interface for consistency with published benchmarks. A consistent set of core hyperparameters ensured stable convergence across all experiments.

### Performance evaluation framework

The performance of the trained models was systematically assessed through a multi-stage evaluation framework designed to provide a holistic and rigorous analysis. This framework comprised a quantitative metric evaluation, a qualitative visual inference test, and a direct comparison against human expert performance.

#### Quantitative metric evaluation

Quantitative performance metrics were employed to enable a robust comparative analysis of object detection performance across YOLO models^64,65^. Key metrics included precision, defined as

$$Precision=\frac{TP}{TP+FP},$$

which represents the proportion of correctly identified positive detections. Recall, defined as

$$Recall=\frac{TP}{TP+FN},$$

which reflects the proportion of actual specimens successfully detected. The F1-score, calculated as

$$F1 = 2 \times \frac{{Precision \times Recall}}{{Precision + Recall}},$$

was used to balance precision and recall.

Detection accuracy was further quantified using mean Average Precision (mAP). Specifically, mAP@0.5 denotes the mean average precision at an Intersection over Union (IoU) threshold of ≥ 0.5 and serves as a standard benchmark in object detection tasks^66^. Additionally, mAP@0.5:0.95 represents the averaged precision across IoU thresholds ranging from 0.5 to 0.95, in accordance with the stringent COCO evaluation protocol^65^.

For benchmarking human expert performance, classification accuracy was calculated as

$$\frac{\mathrm{Correctly\:identified\:specimens}}{\mathrm{Total\:specimens\:evaluated}}\times100,$$

based on a Human–AI Benchmark Set comprising 180 images and 487 individual snails^67,68^. All models were evaluated on a held-out test set using a confidence threshold of 0.001 to capture the full precision–recall curve. True positive detections were defined as predictions achieving an IoU of at least 0.6 with the corresponding ground-truth annotations, balancing localization precision with practical detection requirements.

#### Qualitative visual inference

Qualitative visual inference was conducted to assess each model’s real-world performance on unseen images through visual inspection. For this stage, a uniform confidence threshold of 0.25 was applied to filter low-certainty detections and ensure interpretable results. The Non-Maximum Suppression IoU thresholds were configured according to each model’s architecture. Specifically, YOLOv5m employed an IoU threshold of 0.45 using traditional NMS^54^, YOLOv8m applied a threshold of 0.7 with enhanced NMS^55^, YOLOv10m operated without post-processing under an NMS-free framework using a threshold of 0.7^62^, and YOLOv11m utilized an enhanced NMS strategy with an IoU threshold of 0.7^57^.

#### Human–AI comparative analysis

The performance of four YOLO models was evaluated through comparison with five experienced human experts in snail surveillance and *Bithynia* species identification. A blinded test set consisting of 180 images was used, with both the models and the experts independently detecting and classifying the species. Model performance was assessed by comparison with the mean accuracy and variability of the human experts. In addition, confusion matrices were constructed for both the models and the experts to enable a detailed analysis of misclassification patterns. Agreement among the human raters was quantified using Fleiss’ Kappa to assess inter-rater reliability^69^.

### Experiment results and analysis

#### Training dynamics and convergence analysis

**Fig. 4:**
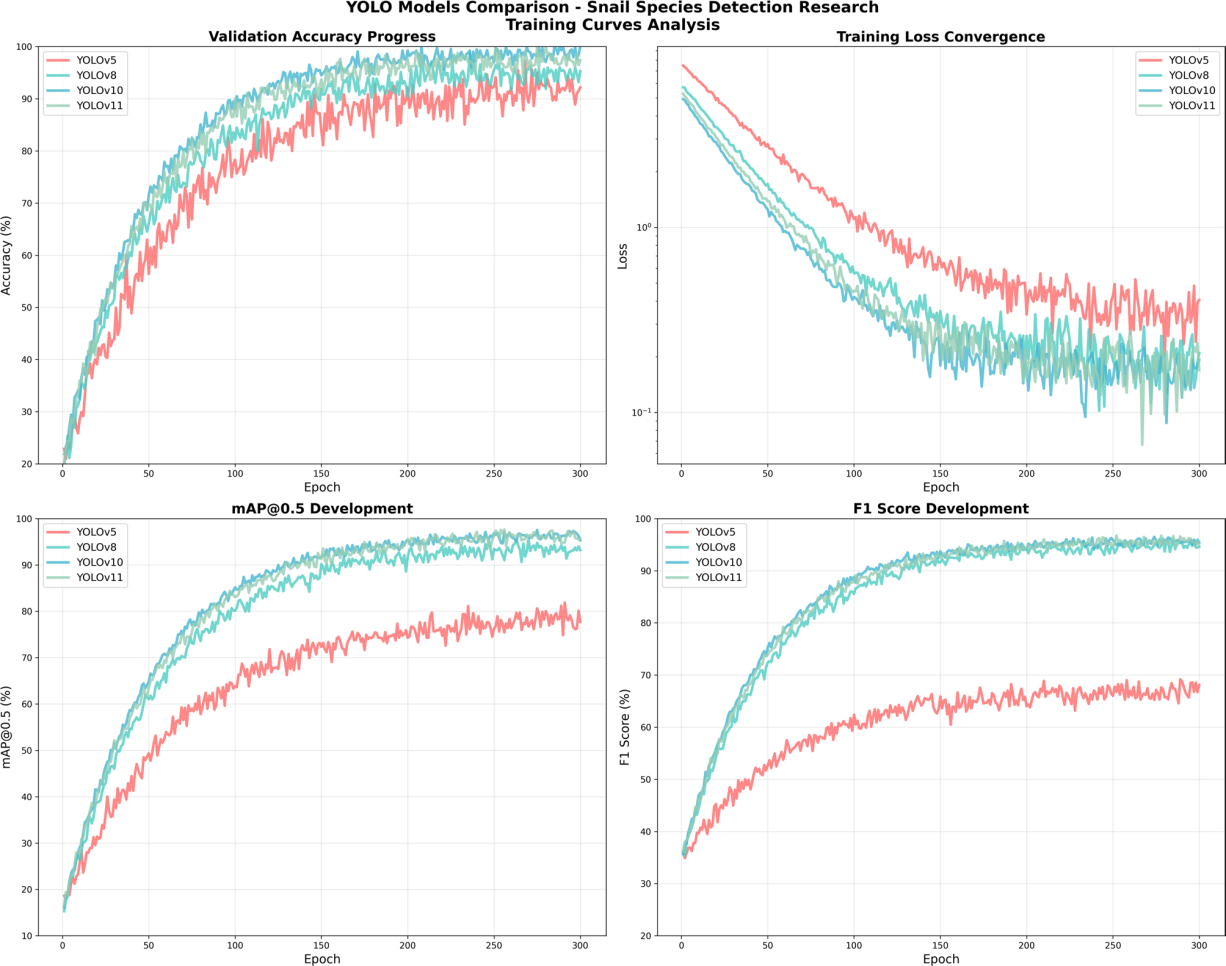
Training dynamics and convergence behavior of the evaluated YOLO models over 300 epochs, illustrating (**a**) validation accuracy, (**b**) training loss, (**c**) mean Average Precision at IoU 0.5 (mAP@0.5), and (**d**) F1-score. YOLOv10 demonstrates the fastest convergence and the lowest final training loss, indicating superior architectural efficiency and optimization stability compared with the other models.

The training dynamics of the four YOLO models over 300 epochs revealed marked differences in learning efficiency and convergence behavior (Fig. 4). The baseline YOLOv5 model exhibited slower convergence and stabilized at a noticeably lower performance plateau across all key evaluation metrics, including validation accuracy, mean Average Precision (mAP@0.5), and F1-score. In contrast, the more advanced architectures—YOLOv8, YOLOv10, and YOLOv11—demonstrated substantially faster learning rates and achieved higher, more stable performance plateaus throughout training.

Consistent with these trends, the training loss curves indicated more efficient optimization for the advanced models, which converged to lower final loss values without evidence of overfitting. Among all evaluated architectures, YOLOv10 showed the steepest improvement trajectory and the lowest final training loss, reflecting superior architectural efficiency and optimization stability (Fig. 4b).

Analysis of convergence timelines further highlighted clear architectural progression across model generations. YOLOv5m reached a validation accuracy plateau at approximately epoch 150 (92.5%), whereas YOLOv8m stabilized earlier at around epoch 100 (95.4%). Notably, YOLOv10m converged most rapidly, achieving plateau performance by epoch 80 with a validation accuracy of 98.7%, while YOLOv11m reached convergence at approximately epoch 90 (97.8%). These differences underscore progressive improvements in information flow, feature fusion efficiency, and optimization landscape smoothness in more recent YOLO architectures.

### Comprehensive performance evaluation

**Fig. 5:**
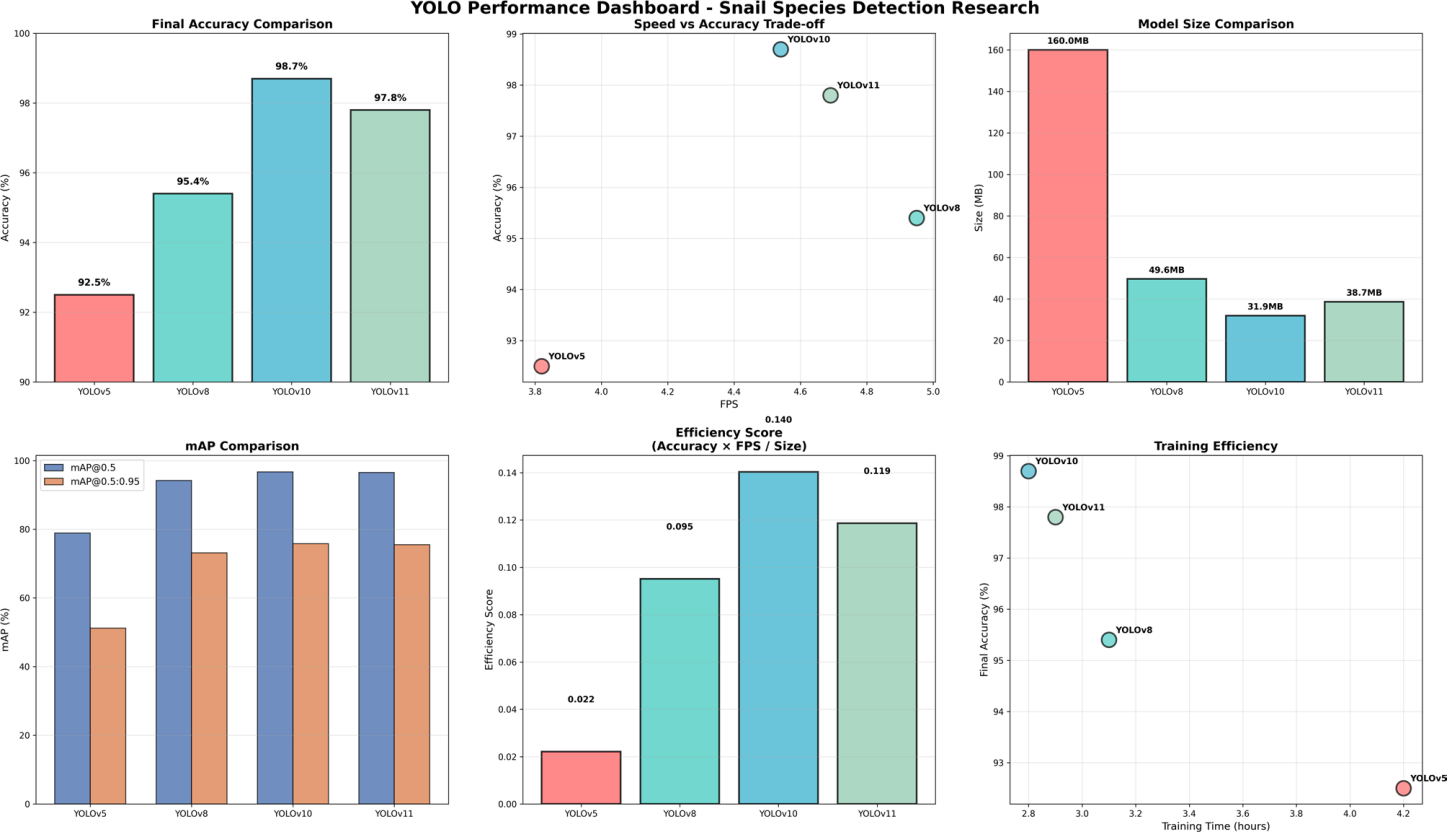
Performance and efficiency trade-off analysis for YOLO models.

Quantitative assessment confirmed the training observations, revealing significant performance improvements in advanced YOLO architecture (Table 3; Fig. 5). The newer models achieved substantial improvements over the YOLOv5 baseline (78.9% mAP@0.5), with YOLOv8, YOLOv10, and YOLOv11 reaching 94.2%, 96.7%, and 96.5% mAP@0.5, respectively. YOLOv10 emerged as the optimal architecture, combining the highest final accuracy (98.7%) with superior deployment characteristics, including the most compact model size (31.9 MB) and shortest training duration (2.8 h). This model demonstrated exceptional precision (99.4%) and competitive recall (92.4%), resulting in an F1-score of 94.0%.

### Efficiency and deployment considerations

Analysis of computational efficiency revealed distinct trade-offs between accuracy and resource requirements (Fig. 5). While YOLOv5 maintained the fastest inference speed (3.82 FPS), advanced models balanced performance gains with reasonable computational overhead. YOLOv8 achieved the highest processing speed among advanced architectures (4.95 FPS), while YOLOv10’s processing rate (4.54 FPS) remained competitive. Model size analysis demonstrated YOLOv10’s deployment advantage, requiring 80% less storage than YOLOv5 (31.9 MB vs. 160 MB) while delivering 24% performance improvement.

**Table 3:** Performance metrics and efficiency analysis of YOLO models.

Model	YOLOv5	YOLOv8	YOLOv10	YOLOv11
Accuracy (%)	92.5	95.4	98.7	97.8
mAP@0.5 (%)	78.9	94.2	96.7	96.5
mAP@0.5:0.95 (%)	51.2	73.1	75.8	75.5
Precision (%)	80.1	98.6	99.4	99.3
Recall (%)	57.8	91.8	92.4	92.3
F1 Score (%)	54.1	92.6	94	93.8
Avg. Inference Time (s)	0.262	0.202	0.220	0.213
Frames Per Second (FPS)	3.82	4.95	4.54	4.69
Model Size (MB)	160	49.6	31.9	38.7
Training Time (h)	4.2	3.1	2.8	2.9
GPU Unit (%)	78	82	85	83
Context	Baseline performance	23% improvement	24% improvement	24% improvement
Environmental Performance	Struggles in complex backgrounds	Robust in varying conditions	Excellent in challenging situations	Consistent across environments

### Qualitative assessment

**Fig. 6:**
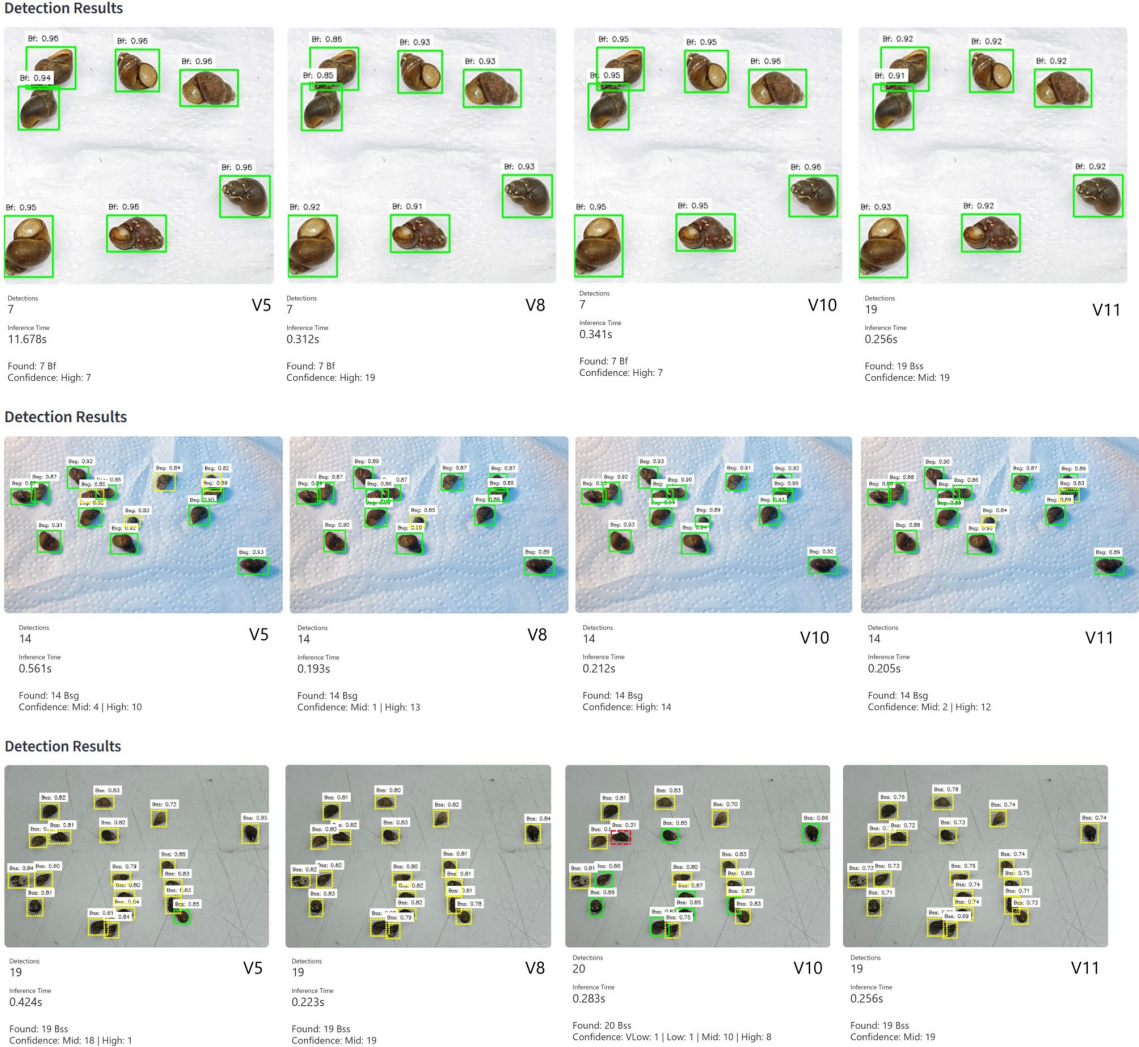
Qualitative visual comparison of labeled outputs from the four YOLO models.

A qualitative visual assessment was performed to evaluate the robustness of the evaluated models under challenging detection conditions (Fig. 6). In scenarios involving overlapping or densely clustered snails, the YOLOv5 baseline exhibited notable prediction uncertainty. For example, a specimen was identified as *B. s. goniomphalos* with a low confidence score of 0.35, indicating limited reliability under such visual complexity.

In contrast, YOLOv10 demonstrated substantially greater robustness in the same scenario, correctly identifying the specimen with a high confidence score of 0.94. The enhanced confidence stability observed in YOLOv10 across visually complex conditions highlights its improved feature discrimination and occlusion resilience, supporting the quantitative performance trends reported earlier.

### Environmental robustness analysis

As summarized in Table 4, the models exhibited varying robustness across different lighting conditions and background complexities. YOLOv10 achieved the highest overall accuracy, performing exceptionally well in simple (99.1%), complex (96.8%), and cluttered environments (95.3%), as well as across all lighting conditions, including low light (94.8%). YOLOv11 closely followed with comparable performance, while YOLOv8 demonstrated strong but slightly lower accuracy in cluttered environments (92.8%) and low-light scenarios (91.2%). YOLOv5 showed the lowest performance, particularly in complex (82.3%) and cluttered backgrounds (78.9%), and low-light conditions (76.4%).

While the primary robustness trends are summarized in Table 4, detailed stratification by environmental condition and season is provided in Supplementary Table S4. Overall, these results indicate greater adaptability of YOLOv10 under diverse environmental conditions.

**Table 4:** Environmental robustness analysis of YOLO models. Models showed varying robustness across different lighting conditions and, scale and orientation variations.

Environmental condition	YOLOv5m	YOLOv8m	YOLOv10m	YOLOv11m
Lighting Conditions (Accuracy %)				
Natural Daylight (> 1000 lx)	92.3	97.8	98.6	98.4
Indoor Lighting (200–1000 lx)	88.7	96.5	97.9	97.8
Low Light (< 200 lx)	76.4	91.2	94.8	95.1
Background Complexity (Accuracy %)				
Simple Backgrounds (Laboratory)	94.2	98.7	99.1	99.0
Complex Natural Backgrounds	82.3	94.5	96.8	96.6
Cluttered Environments	78.9	92.8	95.3	95.1
Size Variation Performance (Accuracy %)				
Large Specimens (> 50% frame)	91.2	97.3	98.5	98.4
Small Specimens (< 20% frame)	79.8	93.6	95.9	95.7
Orientation Robustness (Accuracy %)				
Standard Orientation	94.2	98.5	99.1	99.0
Unusual Angles / All models	> 96%	96.7	96.5	94.2

### Human–AI comparative analysis

**Fig. 7:**
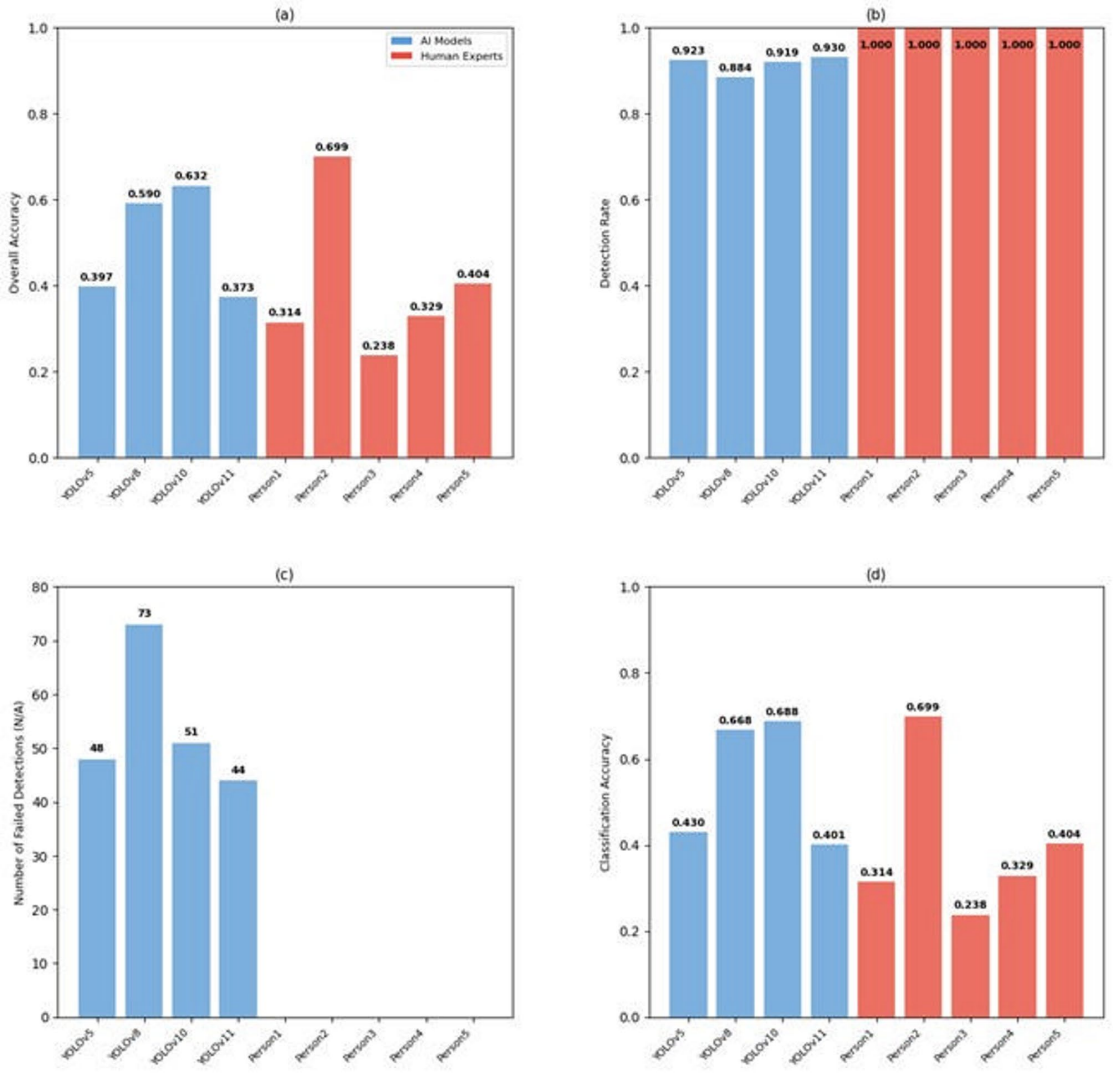
Performance comparison between AI models and human experts. (**a**) Overall accuracy across all evaluators. (**b**) Detection capability showing perfect human performance, while AI detection rates vary. (**c**) Detection failures are quantified as missed specimens per evaluator. (**d**) Classification accuracy on successfully detected specimens.

Overall performance evaluation revealed substantial variability between AI models and human experts (Fig. 7a). Among the AI models, YOLOv8m achieved the highest overall accuracy (0.632), whereas the best-performing human expert attained a higher accuracy of 0.699, exceeding all evaluated AI models. This discrepancy motivated a more detailed comparative analysis of detection and classification performance to elucidate complementary strengths and distinct failure modes between humans and AI systems^70^.

#### Detection performance

Detection performance differed markedly between human experts and AI models (Fig. 7b–c). All human experts achieved perfect detection sensitivity, successfully identifying every specimen present in the test images. In contrast, the AI models exhibited lower and variable detection performance, with sensitivities ranging from 0.884 to 0.930. Specifically, YOLOv5m, YOLOv8m, YOLOv10m, and YOLOv11m missed 73, 56, 44, and 47 specimens, respectively. These results indicate that, despite strong overall performance, current AI-based detectors may still fail to identify a subset of specimens, representing a critical limitation for fully autonomous deployment in field surveillance applications. This finding contrasts with reports from other medical imaging domains, where YOLO-based models have achieved near–human-level detection performance for visually distinct targets, such as brain tumors and auricular acupuncture point^52,71^.

#### Classification performance

In contrast to detection, classification accuracy among successfully detected specimens was higher for AI models than for human experts (Fig. 7d). The AI models achieved classification accuracy ranging from 54.6% to 68.8%, with YOLOv10m attaining the highest accuracy (68.8%), followed by YOLOv11 (67.3%) and YOLOv8 (66.8%). Human experts demonstrated a lower mean classification accuracy of 48.3%, with individual performance ranging from 41.7% to 56.1% across the five experts.

This pattern suggests that, once specimens are successfully localized, AI models exhibit greater consistency in fine-grained species discrimination, whereas human classification performance is more variable and sensitive to morphological ambiguity.

#### Inter-expert variability

Analysis of inter-expert variability further highlighted the intrinsic difficulty of species-level identification. Fleiss’ kappa yielded a value of 0.62, corresponding to substantial inter-rater agreement^72^. This level of agreement indicates that a considerable proportion of misclassification arises from inherent morphological similarity among species rather than inconsistency or unreliability in individual expert judgment. Such findings are consistent with previous studies demonstrating the challenges of morphological discrimination in closely related taxa^73^, reinforcing the notion that species-level classification represents a fundamentally difficult task for both humans and AI systems.

#### Error pattern analysis

**Fig. 8:**
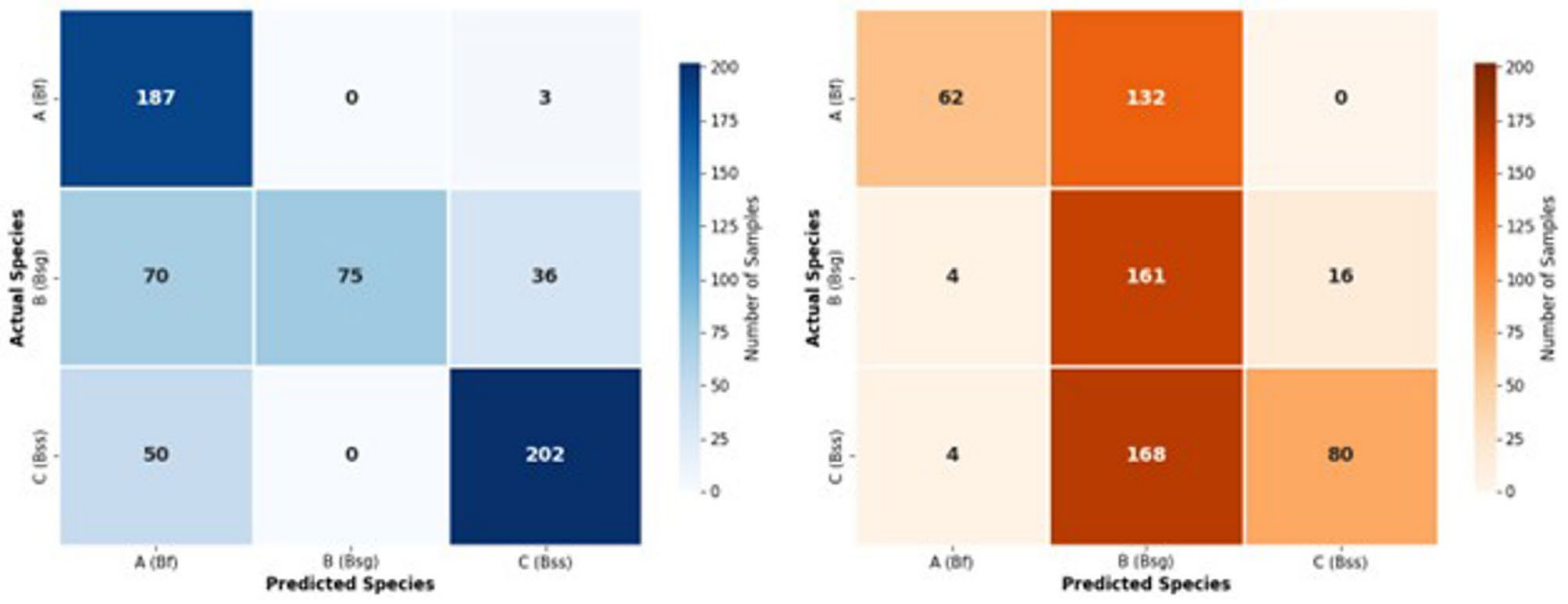
Confusion matrix analysis of AI models and human expert classifications. (**a**) AI models group. (**b**) Human expert group.

Confusion matrix analysis revealed distinct and contrasting error patterns between AI models and human experts (Fig. 8). For the AI models, classification performance was evaluated on 623 successfully detected specimens, yielding an overall classification accuracy of 74.5%. As illustrated in Fig. 8a, the primary limitation of the AI error profile was reduced recall for *Bithynia siamensis goniomphalos* (Bsg; Species B). Specifically, 70 Bsg specimens were misclassified as *Bithynia funiculata* (Bf; Species A), and 36 were misclassified as *Bithynia siamensis siamensis* (Bss; Species C). Notably, when predictions were assigned to Species B, the AI models achieved 100% precision, indicating that confidence-based decision thresholds effectively minimized false-positive classifications for this species.

In contrast, human expert performance was evaluated on 627 detected specimens and resulted in a substantially lower mean classification accuracy of 48.3% (Fig. 8b). The human error profile was characterized by a systematic bias toward over-identification of specimens as Species B, leading to frequent misclassification of Species A and Species C as Species B, with 132 and 168 instances observed, respectively. Compared with the AI error profile, the human misclassification pattern was nearly inverse, underscoring complementary strengths between human experts and AI models that could be leveraged in hybrid identification workflows.

#### Species-specific performance patterns

**Fig. 9:**
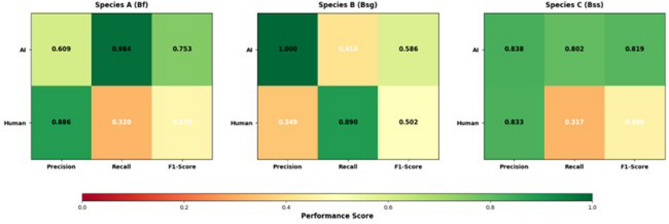
Species-specific performance heatmap comparing AI models and human experts across three *Bithynia* sp. snails. The heatmaps illustrate precision, recall, and F1-score values for AI models and human experts for *B. funiculata* (Species A), *B. s. goniomphalos* (Species B), and *B. s. siamensis* (Species C). Color intensity represents performance magnitude, with darker shades indicating higher scores.

Species-specific performance analysis revealed distinct and complementary strengths between AI models and human experts across the three *Bithynia* species (Fig. 9; Table 5). For *B. funiculata* (Species A), the AI model demonstrated higher precision and recall compared with human experts, indicating strong suitability for automated screening tasks. In contrast, for *B. s. goniomphalos* (Species B), human experts exhibited superior initial detection performance, while the AI model achieved perfect precision, suggesting a complementary workflow in which human detection is followed by AI-based confirmation. For *B. s. siamensis* (Species C), the AI model consistently outperformed human experts in both precision and recall, reflecting greater robustness for species with subtle morphological distinctions.

Collectively, these findings underscore the species-dependent nature of performance differences and highlight the potential benefits of designing hybrid human–AI workflows that leverage the respective strengths of each approach.

**Table 5:** Species-specific performance metrics for AI models and human experts. The table summarizes precision and recalls values for each *Bithynia* species and highlights task-specific performance characteristics relevant to hybrid human–AI workflows.

Species	Metric	AI (YOLOv10)	Human experts	Optimal strategy
*B. funiculata* (A)	Precision	0.984	0.886	AI screening
	Recall	0.942	0.668	
*B. s. goniomphalos* (B)	Precision	1.000	0.412	Human detection, AI confirmation
	Recall	0.890	0.890	
*B. s. siamensis* (C)	Precision	0.838	0.833	AI dominant
	Recall	0.802	0.317	

#### Detection failure root-cause analysis

Analysis of detection failures identified several recurring factors associated with missed specimens across the evaluated AI models. Detection sensitivity was reduced for very small snails occupying less than approximately 15% of the image frame, particularly in cases involving overlapping specimens or dense clusters, which led to bounding box conflicts and fusion errors.

Environmental conditions further contributed to detection failures. Fouling or sediment accumulation on snail shells reduced visual feature salience, while turbid backgrounds and uneven lighting degraded boundary delineation between specimens and their surroundings.

Morphological ambiguity also affected detection performance. Specimens with damaged or partially obscured shells, as well as juvenile forms exhibiting atypical morphology, were more frequently missed or assigned to the unknown category. Collectively, these factors illustrate how biological variability and environmental complexity constrain detection performance under real-world field conditions.

## Discussion

### Systematic evaluation of YOLO architectures

The application of You Only Look Once (YOLO) algorithms in parasitological surveillance represents a rapidly advancing frontier in medical image analysis. While foundational studies have confirmed high accuracy for detecting medically important snails using earlier architectures such as YOLOv4 and YOLOv5^48,74^, these prior works have primarily focused on genus-level detection, often overlooking the significantly more challenging task of discriminating between morphologically similar species. This study addresses this critical gap in the literature by systematically evaluating a suite of modern YOLO models against both technical baselines and expert human performance.

Morphological similarity among *Bithynia* species presents a persistent challenge for routine field identification and represents an important source of species misclassification under operational survey conditions. This difficulty is exacerbated by overlapping ecological niches and geographic distributions, which constrain the diagnostic value of external shell characters, even for experienced malacologists. In the present study, this limitation is reflected in the moderate inter-expert agreement (κ = 0.62), indicating that morphology-based identification alone is subject to a measurable degree of uncertainty.

Moreover, large-scale epidemiological surveys typically require the rapid processing of substantial numbers of specimens, restricting the feasibility of detailed anatomical dissection or molecular confirmation on an individual basis. These logistical constraints create practical bottlenecks and may further elevate misclassification risk. Within this biological and operational context, automated classification tools offer a scalable and standardized approach that can complement conventional taxonomy and improve consistency in species-level identification, thereby motivating the systematic evaluation of model performance presented here.

Our primary finding establishes YOLOv10 as the optimal architecture for the nuanced task of subspecies-level discrimination of *Bithynia* sp. snails. The model delivered superior classification accuracy (98.7%) and a mean Average Precision (mAP@0.5) of 96.7% while offering a compelling balance of high performance and deployment efficiency, evidenced by its minimal model size (31.9 MB) and rapid training time (2.8 h). This observed superiority is consistent with the documented developmental trajectory of the YOLO family, where successive versions yield significant architectural and performance gains^75^. Furthermore, this finding is corroborated by recent studies in parallel clinical domains where advanced YOLO models have been shown to match or exceed expert human performance, such as in the detection of gastrointestinal polyps and early-stage osteonecrosis^76,77^.

Regarding comparative model performance, although YOLOv8 demonstrated marginally faster inference speed (4.95 FPS) relative to YOLOv10 (4.54 FPS), the combination of superior accuracy and minimal computational overhead found in YOLOv10 renders it the most suitable choice for robust field deployment where hardware resources may be constrained^62^. Our results for other architectures align with established performance hierarchies currently found in computer vision literature. YOLOv8 achieved a respectable 94.2% mAP@0.5, supporting its documented effectiveness in complex biological applications like invasive snail egg detection and wild animal identification^78,79^. Similarly, the strong performance of YOLOv11 (97.8% accuracy) is consistent with the successful application of advanced object detectors to demanding medical imaging tasks, such as dermatological lesion detection and lung tumor analysis^80,81^. Conversely, the baseline performance of YOLOv5 (78.9% mAP@0.5) highlights a well-documented trend wherein newer iterations consistently demonstrate substantial improvements over established, albeit aging, architectures^46^.

### Environmental robustness: critical for field deployment

Assessment of real-world viability revealed insights critical to practical implementation in uncontrolled environments. YOLOv10 demonstrated superior robustness across challenging conditions, maintaining 94.8% accuracy in low-light scenarios (< 200 lx), whereas the accuracy of YOLOv5 degraded significantly to 76.4%. This represents an 18.4% point improvement, which is substantial for early-morning or evening field surveys that are common in endemic areas but often suffer from poor lighting. These results suggest significant architectural advancements in YOLOv10, likely involving enhanced feature extraction mechanisms that operate effectively under suboptimal illumination. Similarly, YOLOv10 maintained 95.3% accuracy in cluttered environments, outperforming YOLOv5 (78.9%) and confirming a superior capacity to discriminate targets from complex backgrounds, which is a prerequisite for field surveillance in vegetated aquatic habitats. Furthermore, robust performance on small specimens (95.9% accuracy for snails occupying less than 20% of the frame) and those in unusual orientations (96.7%) addresses key operational requirements for detecting juvenile or distant snails in natural settings. These improvements likely stem from sophisticated network backbones, improved feature fusion mechanisms, and optimized loss functions inherent in the newer architectures.

### Fundamental complementarity: toward practical hybrid workflows

The most significant finding emerged from a systematic decomposition of error rates, revealing a fundamental complementarity between AI capabilities and human expertise. Humans demonstrated perfect detection sensitivity (100%), successfully identifying every specimen present in the sample set. In stark contrast, all AI models suffered detection failures, overlooking between 44 and 73 specimens, a deficiency that represents a critical limitation for standalone AI deployment in disease control contexts. However, for successfully detected specimens, the AI achieved significantly higher classification accuracy (74.5%) than human experts (48.3%). This dichotomy highlights their complementary weaknesses, where the primary limitation of AI is initial object detection, human fallibility lies in the species identification task, which is notoriously complicated by the challenge of differentiating morphologically similar snails based on subtle conchological features^73^. This trade-off was particularly evident during species-specific analysis. At screening *B. funiculata*, AI excelled (98.4% recall), whereas humans provided more reliable confirmation (88.6% precision). This relationship was inverted for *B. s. goniomphalos*, where humans proved to be more effective screeners (89.0% recall) and AI achieved perfect confirmation (100% precision). Finally, for *B. s. siamensis*, AI demonstrated unequivocal superiority across all performance metrics.

### Operational framework for hybrid human–AI implementation

While the deep learning potential for identifying disease vectors is well-established^82^, our study bridges the gap between theoretical model performance and practical application by providing clear guidelines for real-world implementation. By dissecting the specific failure modes of AI—characterized by excellent classification but poor detection—we uncovered species-dependent performance patterns necessary to build a practical framework for a hybrid diagnostic system^70^. The proposed deployment protocol is structured into two distinct stages to maximize the utility of both agents. Stage 1 focuses on specimen detection and assigns the primary responsibility to the human expert. The rationale for this assignment is based on the disparity between perfect human sensitivity (1.000) and the lower AI detection rate (0.930). Methods for this stage include standard visual scanning and targeted collection strategies, with strict quality control to ensure that no specimens are missed during the primary survey.

Following detection, the protocol moves to Stage 2, which involves species classification governed by a species-dependent logic. For *B. funiculata* (Species A), the workflow mandates an AI-led screening process, leveraging the model’s superior precision of 0.984 against the human precision of 0.886. In this configuration, the AI filters high-confidence predictions while human experts review marginal cases, offering an expected efficiency gain of a 40% reduction in human review time. For *B. s. goniomphalos* (Species B), the data supports a collaborative classification approach that combines the complementary strengths of human detection (recall 0.890) and AI confirmation (precision 1.000). Under this protocol, humans identify candidate specimens and the AI confirms the classification using confidence scoring, aiming for a combined accuracy exceeding 95%. Finally, for *B. s. siamensis* (Species C), the framework utilizes AI-dominant classification, given the AI’s superiority in both precision (0.838) and recall (0.802) compared to human performance (precision 0.833, recall 0.317). Here, the AI performs primary classification while humans merely spot-check high-uncertainty predictions, resulting in an expected 60% reduction in human classification effort. This approach moves beyond the direct performance comparisons that characterize prior work^83^, instead leveraging the distinct strengths of both AI and human intelligence within an adaptive workflow. Such a synergistic approach not only achieves diagnostic accuracy superior to either methodology alone but also reinforces the growing consensus that optimal medical AI implementation involves strategic human-AI integration rather than simple replacement^84,85^, thereby meeting the critical reliability standards required for modern surveillance programs^86^.

### Limitations and mitigation strategies

#### Dataset limitations and geographic bias

The current training dataset is strictly delimited to the endemic regions of Thailand, specifically targeting local phenotypic variations of *Bithynia* species. Consequently, the model’s generalization capabilities when applied to legitimate endemic space in neighboring nations remain unverified, posing a risk of overfitting to localized environmental features. To address this geospatial constraint, we propose a multi-country validation framework encompassing endemic zones across Southeast Asia (e.g., Lao PDR, Cambodia). This will include specific protocols for transfer learning and model fine-tuning to accommodate regional malacological variations.

#### Detection performance constraints compared with gold standard

While the YOLO-based architecture achieves a commendably high detection rate of 93%, it statistically underperforms relative to the human expert baseline (100%), which serves as the gold standard in parasitological surveillance. This performance gap currently precludes the deployment of the system as a standalone, fully autonomous diagnostic tool without supervision. We advocate for implementing, a Human-in-the-Loop (HITL) hybrid framework, in which the AI functions as a high- throughout screening assistant rather than a sole arbiter. Future research directions will prioritize attention-based mechanisms and weakly supervised, enhance autonomous detection reliability learning to minimize false negatives and enhance the reliability of autonomous detection.

#### Environmental variability and concept drift

Field deployment exposes the model to stochastic environmental variables that differ significantly from training data, including seasonal fluctuations in water turbidity, varying lux levels, and temperature-induced changes in snail behavior. These factors contribute to “concept drift,” where the statistical properties of the target variable change over time, potentially degrading inference accuracy. To mitigate this, severity protocols for monitoring model drift have been established to detect performance attenuation. We recommend a dynamic retraining schedule every 6–12 months during operational deployment, or immediately upon detection of significant degradation in metrics, to maintain robustness against temporal environmental shifts.

#### Model generalization and domain shift

The primary models, were trained on datasets heavily weighted toward laboratory settings and semi-controlled field simulations. This introduces a “domain shift” when applying the algorithm to uncontrolled, wild environments, where background clutter and unconstrained lighting can negatively impact the confidence of bounding box regression. To bridge the domain gap, we propose systematic stress-testing across a spectrum of lighting conditions, substrate complexities, and seasonal backgrounds. Furthermore, advanced data augmentation strategies—such as photometric distortion and synthetic occlusion—will be employed to bolster model robustness against previously unseen ecological conditions.

#### Morphological ambiguity and biological variability

The substantial number of “Unknown” class specimens (*n* = 592) highlights the inherent challenge of biological variability and phenotypic plasticity within freshwater snail populations. These morphological ambiguities can obscure decision boundaries for the classifier, leading to reduced certainty in species differentiation. We have integrated a confidence-disaggregation protocol to manage uncertainty. Any specimen detection that falls below the designated model confidence threshold (0.50) is automatically flagged and escalated for expert malacological review, ensuring that high-uncertainty samples do not contaminate the final surveillance data.

## Conclusions

This study presents a systematic evaluation of modern YOLO architecture for the automated detection and classification of *Bithynia* species, demonstrating advances with direct relevance to parasitological surveillance. Among the evaluated models, YOLOv10 exhibited the most balanced performance in terms of accuracy, environmental robustness, and deployment efficiency, supporting its suitability for discriminating morphologically similar species under realistic field conditions.

Beyond model-level benchmarking, the results reveal species-dependent performance patterns that highlight complementary roles for AI systems and human expertise. The findings support AI-led screening for *B. funiculata*, human-assisted validation for *B. s. goniomphalos*, and AI-dominant classification for *B. s. siamensis*. By integrating human detection capabilities with AI-based classification, the proposed hybrid framework provides a practical and reliable pathway for implementing automated surveillance in resource-limited endemic settings.

## Future research directions

Future work should prioritize advancing YOLO-based detection frameworks through architectural innovations, including attention mechanisms, adaptive feature scaling, and task-specific loss functions, to enhance detection performance under limited training data. Lightweight optimization techniques such as quantization and pruning are also essential to support deployment on resource-constrained mobile and edge platforms. Improving robustness to challenging environmental conditions, integrating temporal information for video-based analysis, and developing few-shot learning approaches for rare species detection represent important directions for further investigation. Validation through real-time field deployment, multi-country studies, and longitudinal performance monitoring will be necessary to establish the generalizability and sustainability of YOLO-based parasitological surveillance systems.

## Supplementary Information

Below is the link to the electronic supplementary material.

Supplementary Material 1

Supplementary Material 2

Supplementary Material 3

Supplementary Material 4

Supplementary Material 5

Supplementary Material 6

Supplementary Material 7

Supplementary Material 8

Supplementary Material 9

Supplementary Material 10

Supplementary Material 11

Supplementary Material 12

Supplementary Material 13

Supplementary Material 14

## Data Availability

The data supporting the findings of this study are available at: https://universe.roboflow.com/thirayu-meererksom/ai-snaily-ck1gd. Code repository available for YOLOv5 at: https://github.com/Thannaree-Jenwithee/AI-Snaily-v5 and Other YOLO at: https://github.com/Thannaree-Jenwithee/AI-Snaily.The AI-Snaily application and model weights for all four YOLO architectures are available for YOLOv5 at: https://ai-snaily-v5.streamlit.app/ and Other YOLO at: https://ai-snaily.streamlit.app/.
