# Supplementary figures and images for: Automated real-time surveillance of Bithynia snails using a comparative YOLO based approach for liver fluke host detection
Source: Sci Rep. 2026 Mar 24;16:14886. doi: 10.1038/s41598-026-43387-x (PMC13168470; doi:10.1038/s41598-026-43387-x)

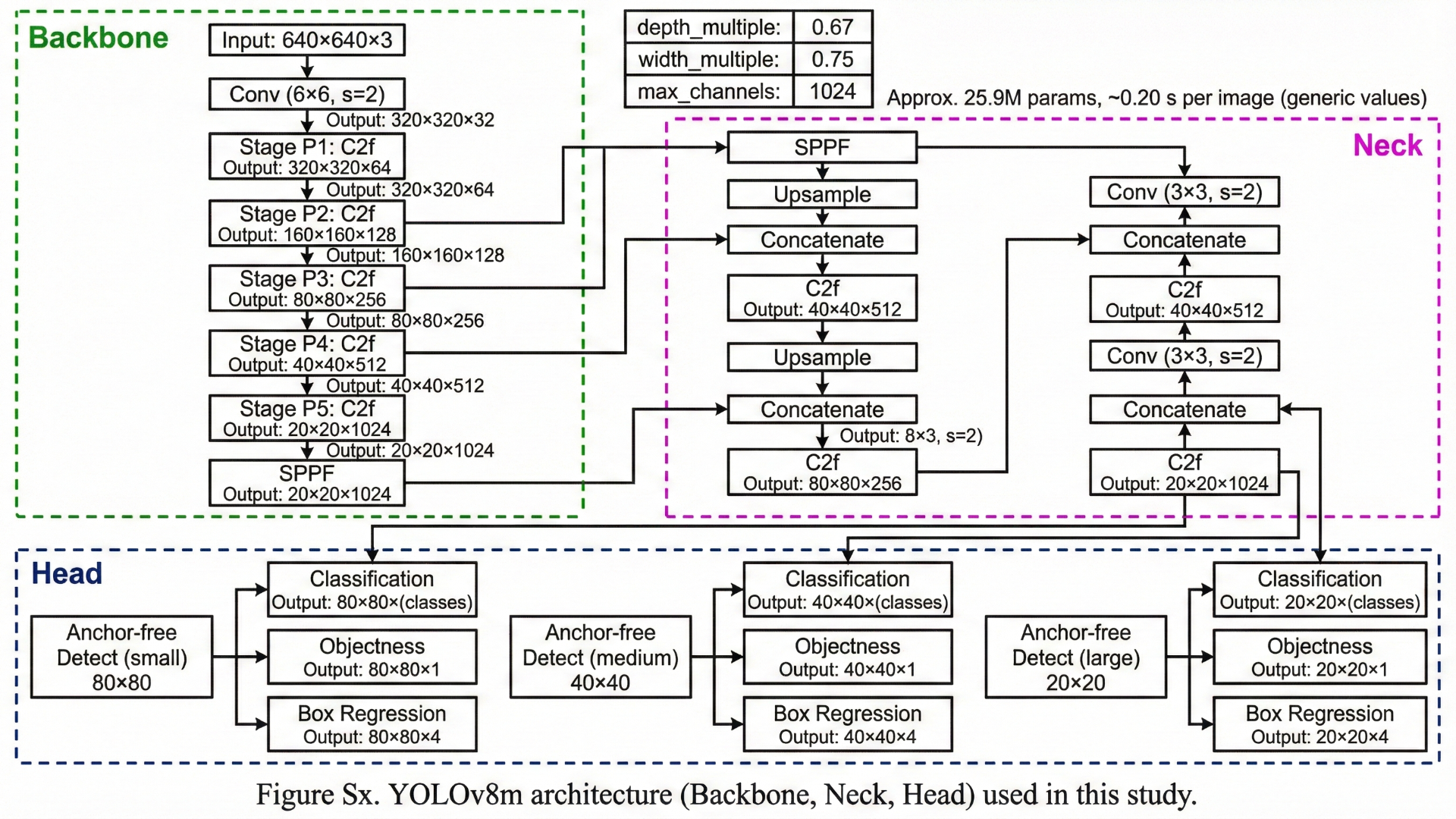

Supplement: Supplementary file 1 — Supplementary Material 1 [file 41598_2026_43387_MOESM1_ESM.tiff]

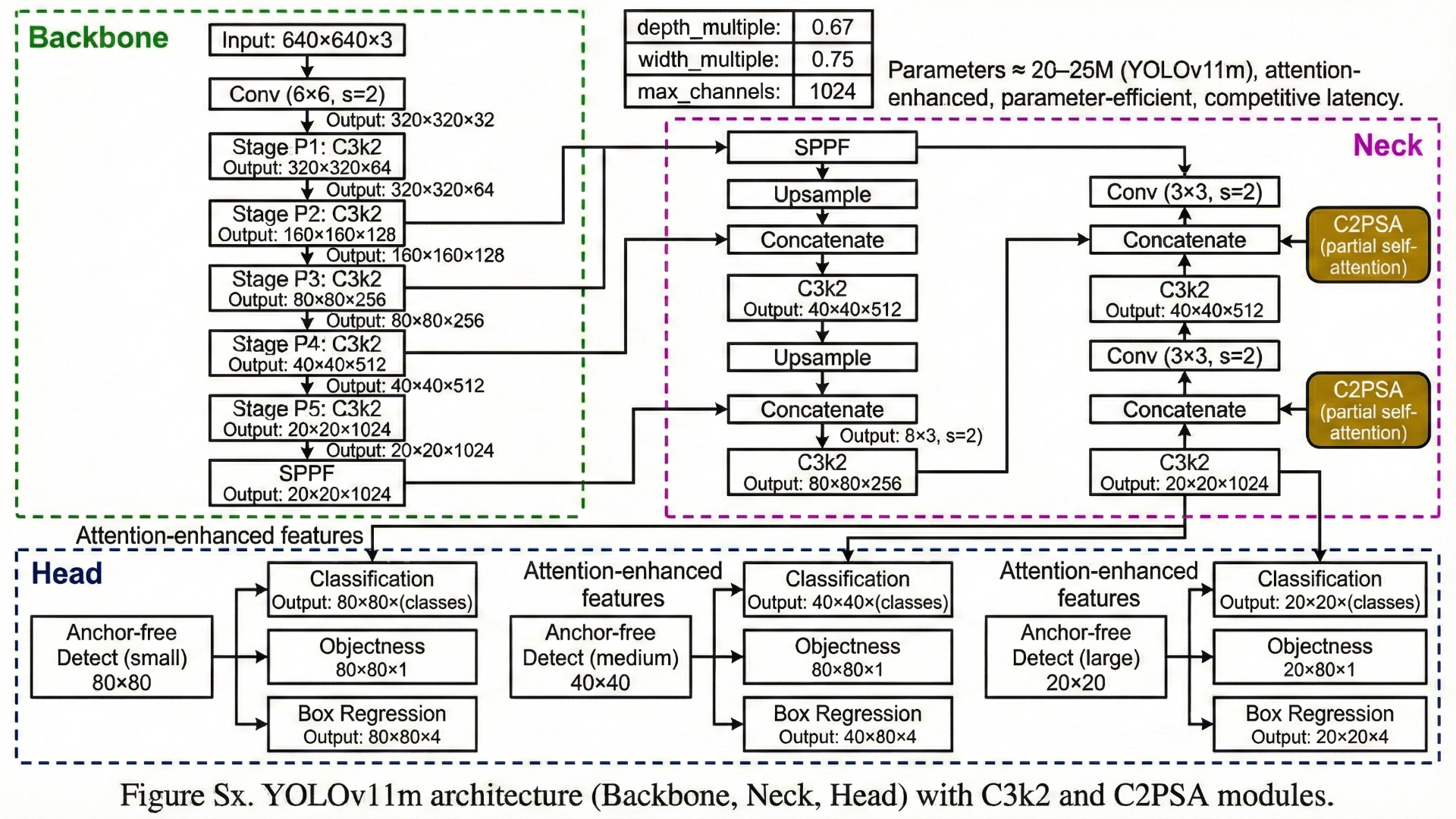

Supplement: Supplementary file 3 — Supplementary Material 3 [file 41598_2026_43387_MOESM3_ESM.tiff]

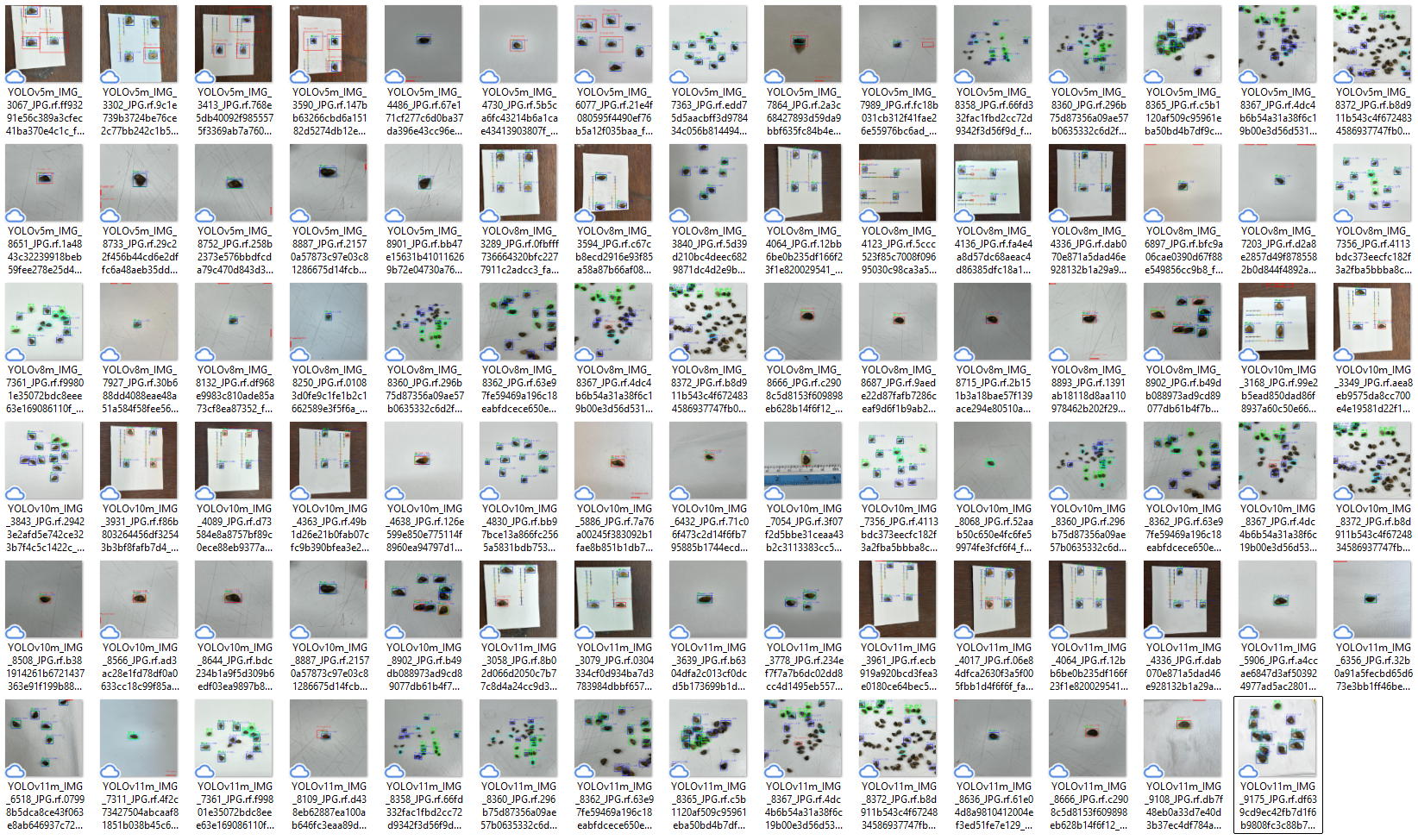

Supplement: Supplementary file 5 — Supplementary Material 5 [file 41598_2026_43387_MOESM5_ESM.tiff]

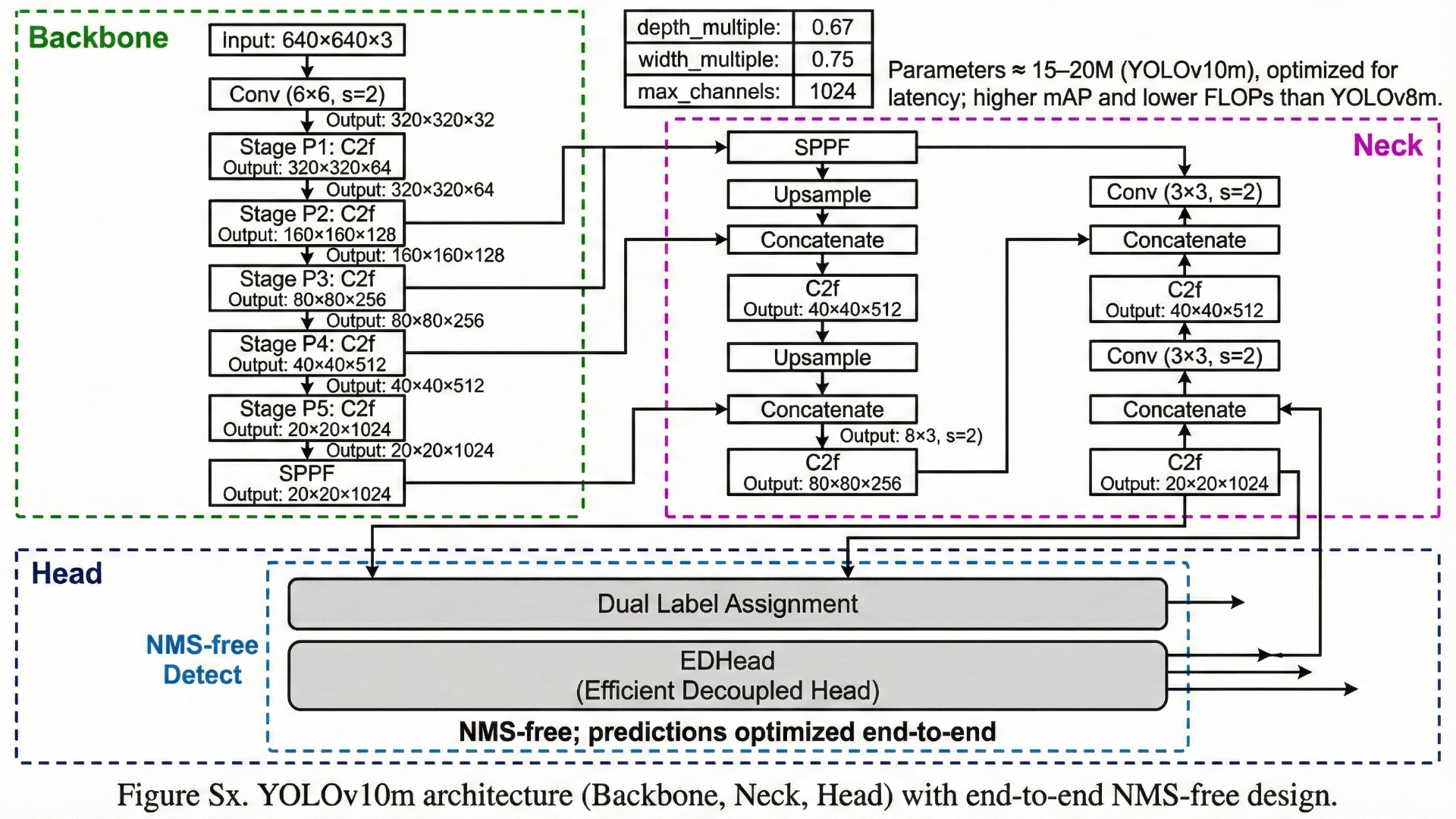

Supplement: Supplementary file 7 — Supplementary Material 7 [file 41598_2026_43387_MOESM7_ESM.tiff]

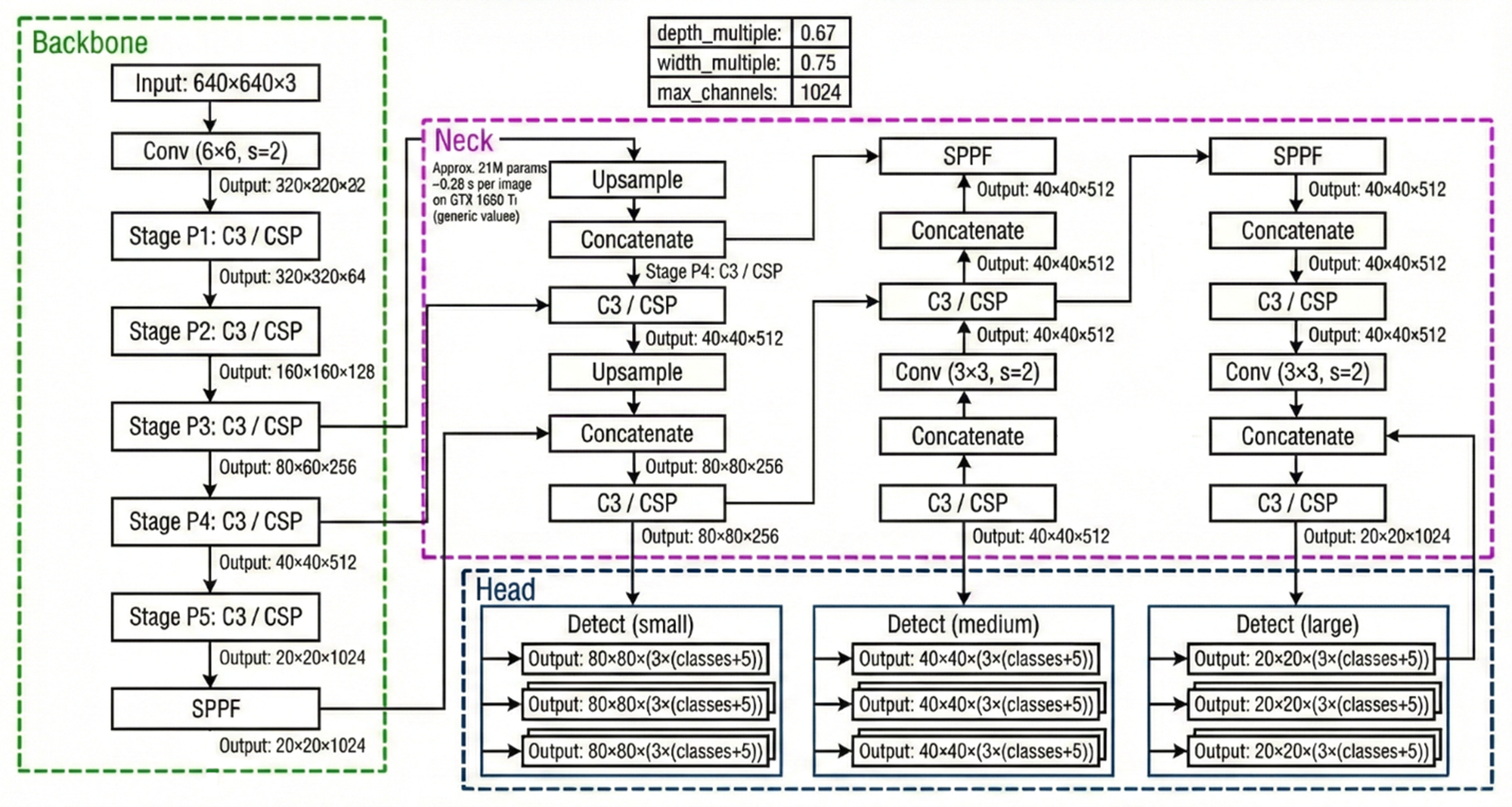

Supplement: Supplementary file 9 — Supplementary Material 9 [file 41598_2026_43387_MOESM9_ESM.tiff]

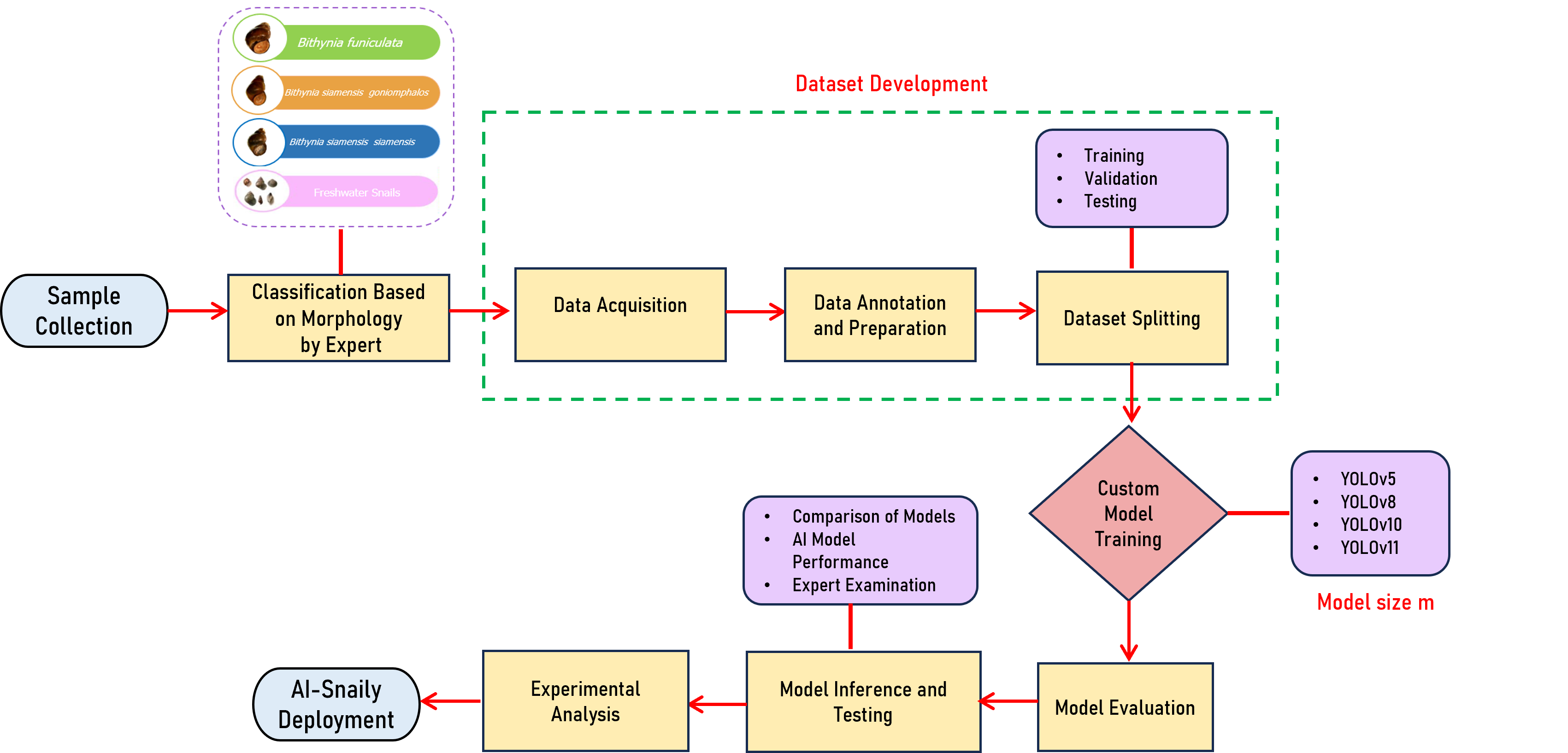

Supplement: Supplementary file 11 — Supplementary Material 11 [file 41598_2026_43387_MOESM11_ESM.tiff]

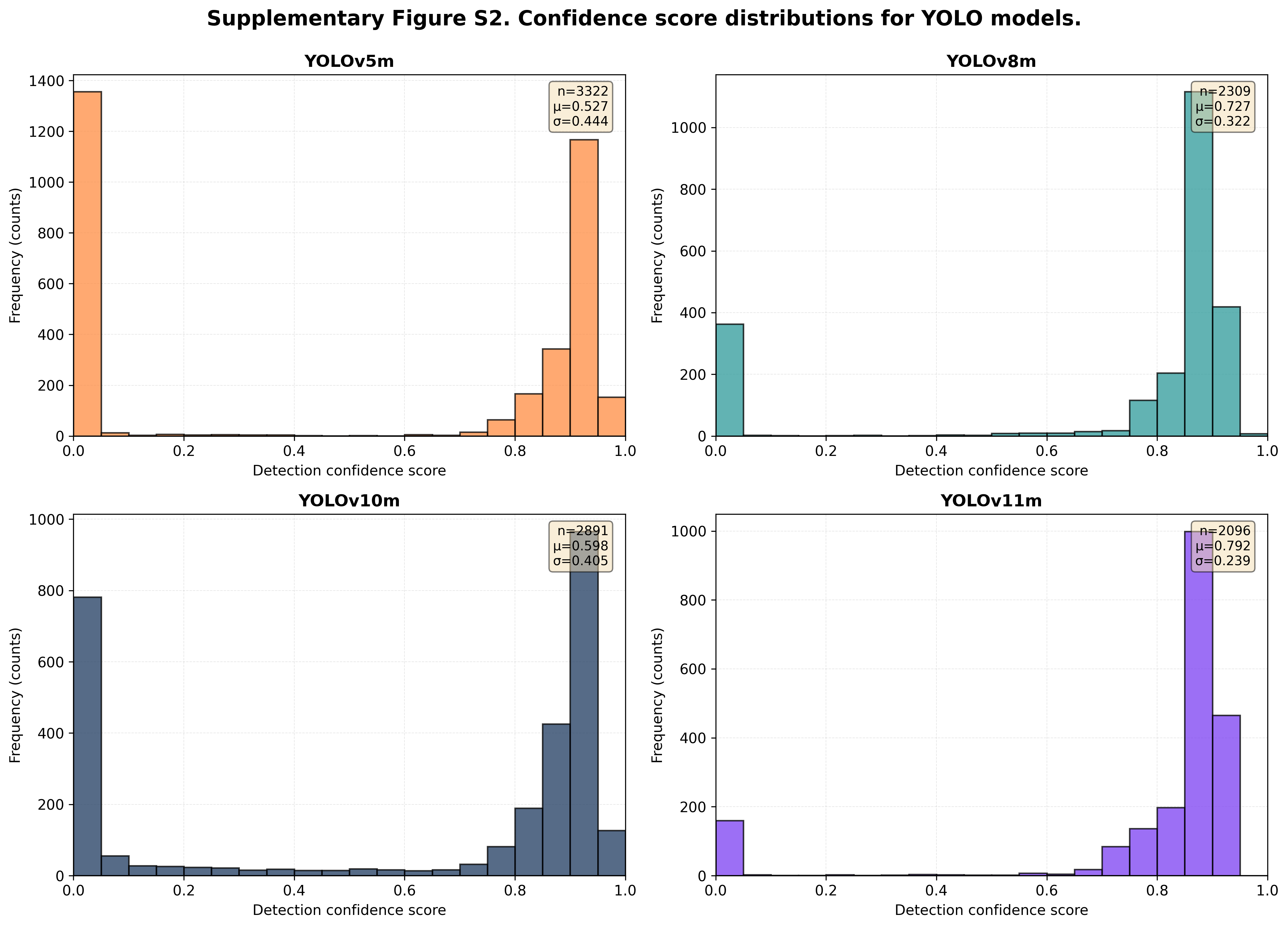

Supplement: Supplementary file 13 — Supplementary Material 13 [file 41598_2026_43387_MOESM13_ESM.tiff]
